# Intensive care treatment in acute pulmonary embolism in Germany, 2016 to 2020: a nationwide inpatient database study

**DOI:** 10.1016/j.rpth.2024.102545

**Published:** 2024-08-08

**Authors:** Karsten Keller, Ingo Sagoschen, Ioannis T. Farmakis, Katharina Mohr, Luca Valerio, Johannes Wild, Stefano Barco, Frank P. Schmidt, Tommaso Gori, Christine Espinola-Klein, Thomas Münzel, Philipp Lurz, Stavros Konstantinides, Lukas Hobohm

**Affiliations:** 1 Department of Cardiology, University Medical Center of the Johannes Gutenberg-University Mainz, Mainz, Germany; 2 Center for Thrombosis and Hemostasis (CTH), University Medical Center of the Johannes Gutenberg-University Mainz, Mainz, Germany; 3 Medical Clinic VII, Department of Sports Medicine, University Hospital Heidelberg, Heidelberg, Germany; 4 Institute of Medical Biometry and Statistics, Faculty of Medicine and Medical Center, University of Freiburg, Freiburg, Germany; 5 Department of Internal Medicine, University Clinic Gießen and Marburg, Marburg, Germany; 6 Department of Angiology, University Hospital Zurich, Zurich, Switzerland; 7 Department of Cardiology, Mutterhaus Trier, Trier, Germany; 8 German Center for Cardiovascular Research (DZHK), Partner Site Rhine Main, Mainz, Germany; 9 Department of Cardiology, Democritus University of Thrace, Alexandroupolis, Greece

**Keywords:** hemodynamic instability, ICU, intensive care, pulmonary embolism, venous thromboembolism

## Abstract

**Background:**

Pulmonary embolism (PE) is a potentially life-threatening condition. Admission and treatment in the intensive care unit (ICU) is an important element in critically ill PE patients.

**Objectives:**

We aimed to identify risk factors for ICU admission and differences in patient profiles regarding risk factors and comorbidities between PE patients who had to be admitted to an ICU and those who were treated in a normal ward without ICU.

**Methods:**

We used the German nationwide inpatient sample to analyze all hospitalizations of PE patients in Germany from 2016 to 2020 stratified for ICU admission.

**Results:**

Overall, 484,859 hospitalized PE patients were treated in German hospitals from 2016 to 2020. Among these, 92,313 (19.0%) were admitted to ICU. Patients treated in ICU were younger (69.0 [IQR, 58.0-78.0] vs 72.0 [IQR, 60.0-80.0] years; *P* < .001) and had higher prevalence of cardiovascular risk factors and comorbidities. In-hospital case fatality rate was elevated in PE patients treated in ICU (22.7% vs 10.7%; *P* < .001), and ICU admission was independently associated with increased in-hospital case fatality (odds ratio [OR], 2.54; 95% CI, 2.49-2.59; *P* < .001). Independent risk factors for ICU admission comprised PE with imminent or present decompensation (OR, 3.30; 95% CI, 3.25-3.35; *P* < .001), hemodynamic instability (OR, 4.49; 95% CI, 4.39-4.59; *P* < .001), arterial hypertension (OR, 1.20; 95% CI, 1.18-1.22; *P* < .001), diabetes mellitus (OR, 1.16; 95% CI, 1.14-1.18; *P* < .001), obesity (OR, 1.300; 95% CI, 1.27-1.33; *P* < .001), surgery (OR, 2.55; 95% CI, 2.50-2.59; *P* < .001), stroke (OR, 2.86; 95% CI, 2.76-2.96; *P* < .001), pregnancy (OR, 1.45; 95% CI, 1.21-1.74; *P* < .001), heart failure (OR, 1.74; 95% CI, 1.71-1.77; *P* < .001), atrial fibrillation/flutter (OR, 1.69; 95% CI, 1.66-1.73; *P* < .001), chronic obstructive pulmonary disease (OR, 1.21; 95% CI, 1.18-1.24; *P* < .001), and renal failure (OR, 1.92; 95% CI, 1.88-1.95; *P* < .001).

**Conclusion:**

ICU treatment is an important element in the treatment of PE patients. Besides hemodynamic compromise, cardiovascular risk factors, stroke, pregnancy, and cardiopulmonary as well as renal comorbidities were independent predictors of ICU admission. Necessity of ICU admission was afflicted by increased case fatality.

## Essentials

• Intensive care unit (ICU) is an important element in the management of pulmonary embolism.
• The German nationwide inpatient sample was used to identify risk factors for ICU admission.
• Hemodynamic compromise and cardiovascular, pulmonary, and renal comorbidities were ICU risk factors.
• Necessity of ICU admission was afflicted by increased case fatality in pulmonary embolism.

## 1 Introduction

Pulmonary embolism (PE) is a major health problem with increasing annual incidence rates ranging between 50 and 100 per 100,000 population [[1], [2], [3]]. PE is a potentially life-threatening condition representing the third most common cardiovascular cause of death after myocardial infarction as well as stroke and is still the leading preventable cause of death in hospitalized patients [[2], [3], [4]]. In Europe, the annual number of PE-related deaths is calculated to exceed 500,000 of the population in a frequently cited epidemiologic model [5,6].

It is well known that depending on clinical severity as well as hemodynamic stability/instability at presentation, more than 16% of the patients suffering acute PE die during the initial hospitalization, and more than 30% may die within the first 30 days [2,3,5,7].

Studies have shown that the case fatality rate is very high in PE patients who need cardiopulmonary resuscitation (CPR) at approximately 84% and in hemodynamically unstable patients with shock but no need for CPR (46.9%) [2,8,9]. In this context, it is of major importance that 30% of all deaths in the entire study population of PE patients and more than 40% of deaths in hemodynamically unstable PE patients occur on the day of admission [2]. Since acute right ventricular (RV) failure as the result of low systemic output is the leading cause of death in patients with high-risk PE (hemodynamic instability) and also in selected patients with threatening hemodynamic compromise (intermediate high-risk), a risk-adapted treatment approach is recommended according to current guidelines for the management of patients with PE [3,5,10,11]. In PE patients who are hemodynamically unstable (high-risk PE), early reperfusion treatment is recommended, whereas in selected normotensive PE patients at risk of imminent decompensation, reperfusion treatments should also be considered as rescue treatment [2,3,5,[10], [11], [12]]. In addition, intensive care unit (ICU) treatment is another important component in adequate management of these hemodynamically compromised PE patients, and ICU capacities can be understood as a bottleneck in adequate PE management. We aimed to identify risk factors for ICU admission and differences in patient profiles regarding risk factors and comorbidities between PE patients who had to be admitted to an ICU and those who were treated in a normal ward without ICU since data in this field of research are sparse.

## 2 Methods

### 2.1 Data source

For this study analysis, we used the German nationwide inpatient sample aiming to investigate temporal trends, risk factors, and the impact of ICU admission in all hospitalization cases of patients with a diagnosis of PE (International Statistical Classification of Diseases and Related Health Problems [ICD] code I26) during the observational period between 2016 and 2020. The statistical analysis of the present study was performed on our behalf by the Research Data Center (RDC) of the Federal Bureau of Statistics (Wiesbaden, Germany). The RDC provided aggregated and summarized statistical results to us on the basis of our created and generated SPSS codes (IBM Corp, released 2011; IBM SPSS Statistics for Windows, Version 20.0, IBM Corp), which we had previously sent to the RDC (source: RDC of the Federal Statistical Office and the Statistical Offices of the federal states, Diagnosis Related Groups Statistics 2016-2020, own calculations) [2].

### 2.2 Study oversight, support, and ethical statement

There was no commercial support and no foreign influence regarding preparation of this manuscript. Since our study did not contain direct access by us (as the study investigators) to individual patient data but only accessed aggregated/summarized results provided by the RDC, approval by an ethics committee as well as patients’ informed consent were both not required in accordance with German law [2].

### 2.3 Coding of diagnoses, procedures, and definitions

A diagnosis- and procedure-related hospital remuneration was introduced in Germany in the year 2004. For this reason, the coding of patient data on diagnoses, coexisting conditions, and surgeries/procedures/interventions according to the German Diagnosis Related Groups system is required for the hospitals to get their remuneration for the rendered and provided services after transfer of these coded data to the Institute for the Hospital Remuneration System [13,14]. For this objective, patients’ diagnoses are coded according to the ICD (10th revision with German modification), and diagnostic/interventional/surgical procedures are coded according to special “Operationen- und Prozedurenschlüssel” (OPS) codes [13,14]. In this German nationwide inpatient sample study, we included all patients with PE (ICD-10 code I26) who were hospitalized in German hospitals from 2016 to 2020 (PE as the main or secondary diagnosis).

### 2.4 Definitions

Obesity was defined according to the recommendations of the World Health Organization as a body mass index of ≥30 kg/m^2^. PE with imminent or present decompensation was defined as tachycardia (ICD-10 codes I47 and R00.0), RV dysfunction (I26.0), or shock (R57). Hemodynamic instability was defined as a shock (R57) or CPR (OPS code 8-77). The following reperfusion treatment procedures were included in the analysis: systemic thrombolysis (OPS code 8-020.8), surgical embolectomy (5-380.42), and catheter-directed thrombolysis or mechanical thrombectomy (8-838.d0, 8-838.50, 8-838.60, 8-838.70, and 8-83b.j). Shock and CPR were defined according to current European guidelines [3,11,15,16].

### 2.5 Study outcomes and adverse in-hospital events

The primary study outcome was admission to ICU. Secondary outcome is case fatality with death of all causes during in-hospital stay (in-hospital case fatality).

### 2.6 Statistical analysis

Descriptive statistics for relevant patient characteristics comparisons of PE patients with and without ICU admission are presented as the median and IQR or absolute numbers and corresponding percentages. We tested the comparisons of continuous variables using the Mann–Whitney U-test and the comparisons of categorical variables with Fisher’s exact or the chi-squared test, as appropriate.

**Figure 1:**
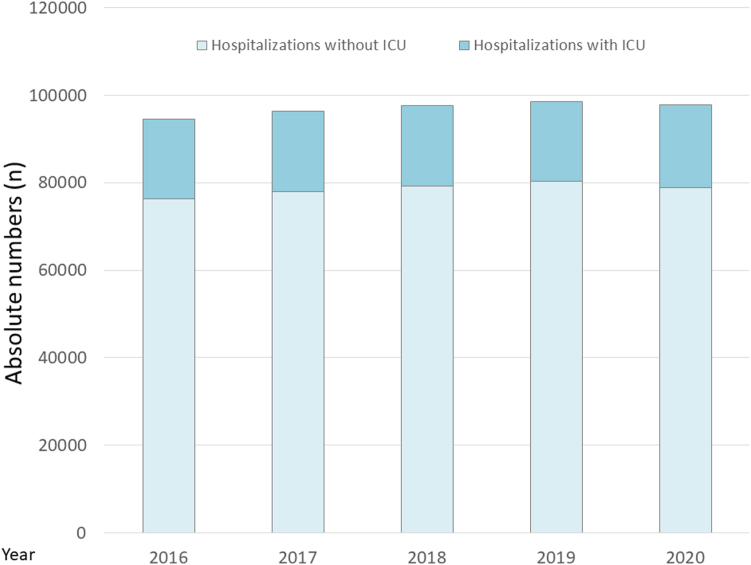
Temporal trends of absolute annual numbers of pulmonary embolism hospitalizations with (dark blue bars) and without intensive care unit (ICU) admission (light blue bars).

Temporal trends regarding the total annual numbers of hospitalizations of PE patients and the proportion of PE patients admitted to ICU are descriptively illustrated in Figure 1.

Univariate and multivariate logistic regression models were analyzed to investigate associations between patients’ characteristics, comorbidities, treatments, and in-hospital adverse events on the one hand and ICU admission on the other hand. In addition, we analyzed the association of ICU admission with prolonged in-hospital stay as well as case fatality. The multivariate regression models were adjusted for age, sex (as the stated gender of the patients), obesity, diabetes mellitus, essential arterial hypertension, cancer, surgery, coronary artery disease, heart failure, atrial fibrillation/flutter, chronic obstructive pulmonary disease, acute and chronic kidney failure, and hyperlipidemia. This epidemiologic approach for the adjustment was selected by us to test the widespread independence of the associations regarding influence of these factors. The results are presented as odds ratios (ORs) and 95% CIs. Regarding the logistic regression models, only *P* values <.05 (2-sided) were considered statistically significant.

All statistical analyses were computed with the SPSS software (IBM Corp, released 2011; IBM SPSS Statistics for Windows, version 20.0).

## 3 Results

Overall, 484,859 hospitalized patients with PE (median age, 71.0 [IQR, 59.0-80.0] years; female sex, 51.0%) were treated in German hospitals during the years 2016 to 2020. Among these, 92,313 (19.0%) were admitted to ICU. The admission rate in ICU was widely stable during the observational period (Figure 1).

Patients treated in ICU were a median 3 years younger (69.0 [58.0-78.0] vs 72.0 [60.0-80.0] years; *P* < .001), more often male (52.9% vs 48.1%; *P* < .001), and had higher prevalence of cardiovascular risk factors as well as comorbidities such as coronary artery disease (16.4% vs 12.5%; *P* < .001), heart failure (34.7% vs 20.6%; *P* < .001), atrial fibrillation/flutter (22.3% vs 12.3%; *P* < .001), and kidney failure (36.6% vs 21.1%; *P* < .001) (Table 1).

**Table 1:** Patients' characteristics, treatments, and outcomes of the 484,859 hospitalized patients with pulmonary embolism during the years 2016 to 2020 in Germany stratified for admission to intensive care unit.

Parameters	PE patients without admission to ICU (*n* = 392,546; 81.0%)	PE patients with admission to ICU (*n* = 92,313; 19.0%)	*P* value
Age (y), median (IQR)	72.0 (60.0-80.0)	69.0 (58.0-78.0)	**<.001**
Age ≥70 y	216,667 (55.2%)	45,210 (49.0%)	**<.001**
Female sex	203,812 (51.9%)	43,505 (47.1%)	**<.001**
In-hospital stay (d)	7.0 (4.0-12.0)	15.0 (8.0-27.0)	**<.001**
Cardiovascular risk factors			
Obesity	32,621 (8.3%)	12,447 (13.5%)	**<.001**
Essential arterial hypertension	180,140 (45.9%)	45,300 (49.1%)	**<.001**
Diabetes mellitus	68,360 (17.4%)	21,558 (23.4%)	**<.001**
Hyperlipidemia	55,564 (14.2%)	14,169 (15.3%)	**<.001**
VTE risk factors			
Cancer	82,825 (21.1%)	19,528 (21.2%)	.71
Any surgery	204,405 (52.1%)	68,762 (74.5%)	**<.001**
Thrombophilia	5185 (1.3%)	1794 (1.9%)	**<.001**
Pregnancy	407 (0.1%)	180 (0.2%)	**<.001**
Comorbidities			
Coronary artery disease	49,169 (12.5%)	15,183 (16.4%)	**<.001**
Heart failure	80,832 (20.6%)	32,054 (34.7%)	**<.001**
Peripheral artery disease	10,868 (2.8%)	3792 (4.1%)	**<.001**
Atrial fibrillation/flutter	48,388 (12.3%)	20,541 (22.3%)	**<.001**
Chronic obstructive pulmonary disease	34,548 (8.8%)	10,898 (11.8%)	**<.001**
Acute and chronic kidney failure	82,668 (21.1%)	33,741 (36.6%)	**<.001**
COVID-19 infection (during the year 2020)	2037 (0.5%)	1325 (1.4%)	**<.001**
Chronic anemia	31,404 (8.0%)	12,502 (13.5%)	**<.001**
Clinical signs of PE severity			
PE with imminent or present decompensation	90,053 (22.9%)	47,137 (51.1%)	**<.001**
Tachycardia	9949 (2.5%)	7187 (7.8%)	**<.001**
Syncope	9504 (2.4%)	2854 (3.1%)	**<.001**
RV dysfunction	80,026 (20.4%)	35,512 (38.5%)	**<.001**
Shock	9427 (2.4%)	16,067 (17.4%)	**<.001**
Hemodynamic instability	21,751 (5.5%)	22,469 (24.3%)	**<.001**
Treatment			
Mechanical ventilation	5851 (1.5%)	13,115 (14.2%)	**<.001**
Systemic thrombolysis	10,595 (2.7%)	9547 (10.3%)	**<.001**
Surgical embolectomy	76 (0.02%)	531 (0.6%)	**<.001**
Catheter-directed treatment	760 (0.2%)	977 (1.1%)	**<.001**
Adverse events during hospitalization			
In-hospital death	42,011 (10.7%)	20,985 (22.7%)	**<.001**
Major adverse cardiac and cerebrovascular events^a^	52,358 (13.3%)	27,122 (29.4%)	**<.001**
Detected deep vein thrombosis and/or thrombophlebitis	142,035 (36.2%)	28,166 (30.5%)	**<.001**
Pneumonia	91,785 (23.4%)	38,575 (41.8%)	**<.001**
Acute kidney failure	23,126 (5.9%)	23,090 (25.0%)	**<.001**
Stroke (ischemic or hemorrhagic)	9513 (2.4%)	7076 (7.7%)	**<.001**
Hemarthrosis	71 (0.02%)	45 (0.05%)	**<.001**
Major bleeding	28,702 (7.3%)	33,183 (35.9%)	**<.001**
Intracerebral bleeding	1466 (0.4%)	1993 (2.2%)	**<.001**
Gastrointestinal bleeding	5322 (1.4%)	3388 (3.7%)	**<.001**
Transfusion of blood constituents	24,072 (6.1%)	31,057 (33.6%)	**<.001**

*P* < .001 are indicated in bold.

ICU, intensive care unit; PE, pulmonary embolism; RV, right ventricular; VTE, venous thromboembolism.

a Defined as all-cause in-hospital death, acute myocardial infarction, or stroke.

All clinical signs of hemodynamic compromise such as shock (17.4% vs 2.4%; *P* < .001) and RV dysfunction (38.5% vs 20.4%; *P* < .001) were more commonly identified in patients with ICU treatment. Consequently, reperfusion treatments of systemic thrombolysis (10.3% vs 2.7%; *P* < .001), catheter-directed treatments (1.1% vs 0.2%; *P* < .001), and surgical embolectomy (0.60% vs 0.02%; *P* < .001) were more often used in PE patients with ICU treatment (Table 1).

**Figure 2:**
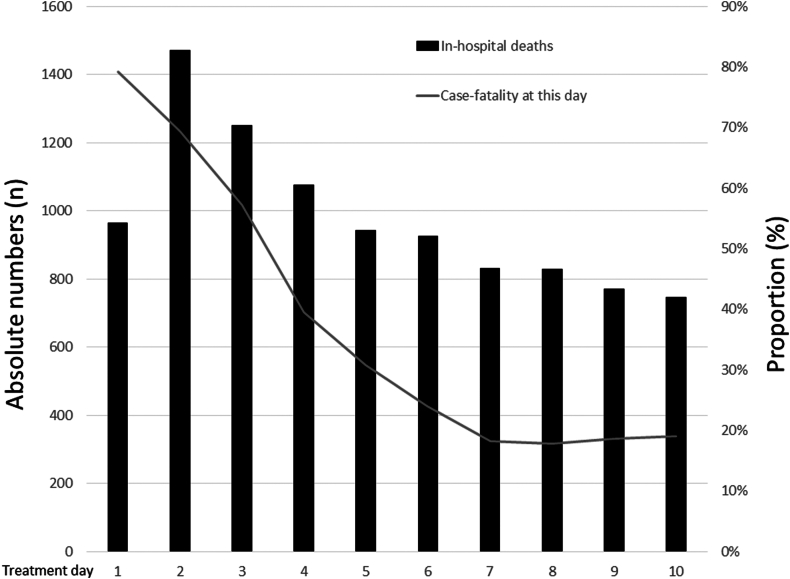
Total number of in-hospital deaths and case fatality rate at the corresponding treatment day of pulmonary embolism patients admitted to an intensive care unit during the first 10 days of hospitalization. The case fatality rate of pulmonary embolism patients at the different treatment days decreased during the illustrated observational period of 10 days.

As expected, the length of in-hospital stay was substantially longer in PE patients with the necessity of ICU treatment (15.0 [8.0-27.0] vs 7.0 [4.0-12.0]; *P* < .001). In addition, the in-hospital case fatality rate was higher in PE patients who had to be treated in ICU vs those without ICU treatment (22.7% vs 10.7%; *P* < .001). The in-hospital deaths of the PE patients treated in an ICU occurred most frequently during the first 4 days of hospitalization (Table 1, Figure 2).

**Figure 3:**
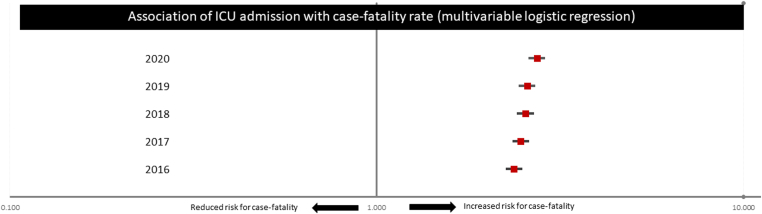
Association of intensive care unit (ICU) admission with case fatality in the different investigated observation years 2016 to 2020.

ICU admission was independently associated with prolonged length of in-hospital stay >10 days (univariate: OR, 4.46; 95% CI, 4.39-4.53; *P* < .001; multivariate: OR, 3.59; 95% CI, 3.53-3.65; *P* < .001) and increased in-hospital case fatality (univariate: OR, 2.46; 95% CI, 2.41-2.50; *P* < .001; multivariate: OR, 2.54; 95% CI, 2.49-2.59; *P* < .001). This association between ICU admission and increased in-hospital death was evident in all investigated years (Figure 3).

Independent risk factors for ICU admission comprise the cardiovascular risk factors of arterial hypertension (OR, 1.20; 95% CI, 1.18-1.22; *P* < .001), diabetes mellitus (OR, 1.16; 95% CI, 1.14-1.18), and obesity (OR, 1.30; 95% CI, 1.27-1.33; *P* < .001), the classical venous thromboembolism (VTE) risk factors such as surgery (OR, 2.55; 95% CI, 2.50-2.59; *P* < .001), stroke (ischemic or hemorrhagic; OR, 2.86; 95% CI, 2.76-2.96; *P* < .001), and pregnancy (OR, 1.45; 95% CI, 1.21-1.74; *P* < .001), and important comorbidities such as heart failure (OR, 1.74; 95% CI, 1.71-1.77; *P* < .001), atrial fibrillation/flutter (OR, 1.69; 95% CI, 1.66-1.73; *P* < .001), chronic obstructive pulmonary disease (OR, 1.21; 95% CI, 1.18-1.24; *P* < .001), acute or chronic renal failure (OR, 1.92; 95% CI, 1.88-1.95; *P* < .001), COVID-19 (OR, 3.26; 95% CI, 3.02-3.52; *P* < .001), and chronic anemia (OR, 1.33; 95% CI, 1.30-1.36; *P* < .001). Consecutively, a higher Charlson Comorbidity Index class was afflicted by increased risk for ICU admission (OR, 1.29; 95% CI, 1.28-1.30; *P* < .001; Table 2).

**Table 2:** Regression analysis of parameters associated with intensive care unit admission in patients with pulmonary embolism.

Parameters	Univariate regression		Multivariable regression^a^	
	OR (95% CI)	*P* value	OR (95% CI)	*P* value
Age	0.99 (0.99-0.99)	**<.001**	0.98 (0.98-0.98)	**<.001**
Age ≥70 y	0.78 (0.77-0.79)	**<.001**	0.58 (0.57-0.59)	**<.001**
Female sex	0.83 (0.81-0.84)	**<.001**	0.88 (0.86-0.89)	**<.001**
Obesity	1.72 (1.68-1.76)	**<.001**	1.30 (1.27-1.33)	**<.001**
Hyperlipidemia	1.10 (1.08-1.12)	**<.001**	0.93 (0.91-0.95)	**<.001**
Arterial hypertension	1.14 (1.12-1.15)	**<.001**	1.20 (1.18-1.22)	**<.001**
Detected deep vein thrombosis and/or thrombophlebitis	0.77 (0.76-0.79)	**<.001**	0.86 (0.85-0.88)	**<.001**
Cancer	1.00 (0.99-1.02)	0.716	0.92 (0.90-0.94)	**<.001**
Surgery	2.69 (2.65-2.73)	**<.001**	2.55 (2.50-2.59)	**<.001**
Pregnancy	1.88 (1.58-2.24)	**<.001**	1.45 (1.21-1.74)	**<.001**
Coronary artery disease	1.38 (1.35-1.40)	**<.001**	1.00 (0.98-1.03)	.79
Heart failure	2.05 (2.02-2.08)	**<.001**	1.74 (1.71-1.77)	**<.001**
Peripheral artery disease	1.50 (1.45-1.56)	**<.001**	1.05 (1.00-1.09)	**.03**
Atrial fibrillation/flutter	2.04 (2.00-2.07)	**<.001**	1.69 (1.66-1.73)	**<.001**
COPD	1.39 (1.36-1.42)	**<.001**	1.21 (1.18-1.24)	**<.001**
Acute or chronic renal failure	2.16 (2.13-2.19)	**<.001**	1.92 (1.88-1.95)	**<.001**
Acute renal failure	5.33 (5.22-5.44)	**<.001**	4.72 (4.59-4.86)	**<.001**
COVID-19	2.79 (2.60-2.99)	**<.001**	3.26 (3.02-3.52)	**<.001**
Diabetes mellitus	1.45 (1.42-1.47)	**<.001**	1.16 (1.14-1.18)	**<.001**
Chronic anemia	1.80 (1.76-1.84)	**<.001**	1.33 (1.30-1.36)	**<.001**
Stroke (ischemic or hemorrhagic)	3.34 (3.24-3.45)	**<.001**	2.86 (2.76-2.96)	**<.001**
Charlson Comorbidity Index	1.08 (1.08-1.09)	**<.001**	-	-
Charlson Comorbidity Index Class	1.29 (1.28-1.30)	**<.001**	-	-
Syncope	1.29 (1.23-1.34)	**<.001**	1.32 (1.27-1.39)	**<.001**
RV dysfunction	2.44 (2.40-2.48)	**<.001**	2.43 (2.39-2.47)	**<.001**
Tachycardia	3.25 (3.15-3.35)	**<.001**	2.52 (2.43-2.60)	**<.001**
PE with imminent or present decompensation	3.51 (3.45-3.56)	**<.001**	3.30 (3.25-3.35)	**<.001**
Hemodynamic instability	5.48 (5.37-5.60)	**<.001**	4.49 (4.39-4.59)	**<.001**
Shock	8.56 (8.34-8.80)	**<.001**	6.23 (6.06-6.41)	**<.001**
Pneumonia	2.35 (2.32-2.39)	**<.001**	2.14 (2.11-2.18)	**<.001**
Systemic thrombolysis	4.16 (4.04-4.28)	**<.001**	4.04 (3.92-4.17)	**<.001**
Surgical embolectomy	29.88 (23.49-38.00)	**<.001**	14.04 (10.97-17.96)	**<.001**
Catheter-directed treatment	5.52 (5.01-6.07)	**<.001**	5.21 (4.71-5.77)	**<.001**
Major bleeding	7.11 (6.99-7.24)	**<.001**	5.61 (5.50-5.73)	**<.001**
Intracerebral bleeding	5.89 (5.50-6.30)	**<.001**	4.87 (4.53-5.23)	**<.001**
Gastrointestinal bleeding	2.77 (2.65-2.90)	**<.001**	2.05 (1.96-2.15)	**<.001**
Transfusion of blood constituents	7.76 (7.62-7.91)	**<.001**	6.10 (5.98-6.23)	**<.001**

*P* < .001 are indicated in bold.

COPD, chronic obstructive pulmonary disease; OR, odds ratio; PE, pulmonary embolism; RV, right ventricular.

a Adjustment level: age, sex, obesity, diabetes mellitus, essential arterial hypertension, cancer, surgery, coronary artery disease, heart failure, atrial fibrillation/flutter, chronic obstructive pulmonary disease, acute and chronic kidney failure, and hyperlipidemia.

As expected, markers of hemodynamic compromise such as PE with imminent or present decompensation (OR, 3.30; 95% CI, 3.25-3.35; *P* < .001) and hemodynamic instability (OR, 4.49; 95% CI, 4.39-4.59; *P* < .001) were independent risk factors for ICU admission (Table 2). Major bleeding was an important reason to be treated in the ICU (OR, 5.61; 95% CI, 5.50-5.73; *P* < .001). All investigated reperfusion treatments were more often used in the ICU (Table 2).

In PE patients admitted to an ICU, the use of systemic thrombolysis (OR, 1.06; 95% CI, 1.01-1.12; *P* = .02), surgical embolectomy (OR, 9.91; 95% CI, 7.56-13.00; *P* < .001), and catheter-directed treatment (CDT) (OR, 1.28; 95% CI, 1.11-1.49; *P* < .001) were independently associated with increased risk of major bleeding. While systemic thrombolysis (OR, 2.23; 95% CI, 2.13-2.35; *P* < .001) and surgical embolectomy (OR, 1.38; 95% CI, 1.13-1.69; *P* = .002) were not independently associated with reduced case fatality in PE patients admitted to the ICU regardless of PE severity, CDT was related to reduced case fatality in PE patients admitted to the ICU (OR, 0.80; 95% CI, 0.67-0.96; *P* = .01) independently of age, sex, and comorbidities.

**Figure 4:**
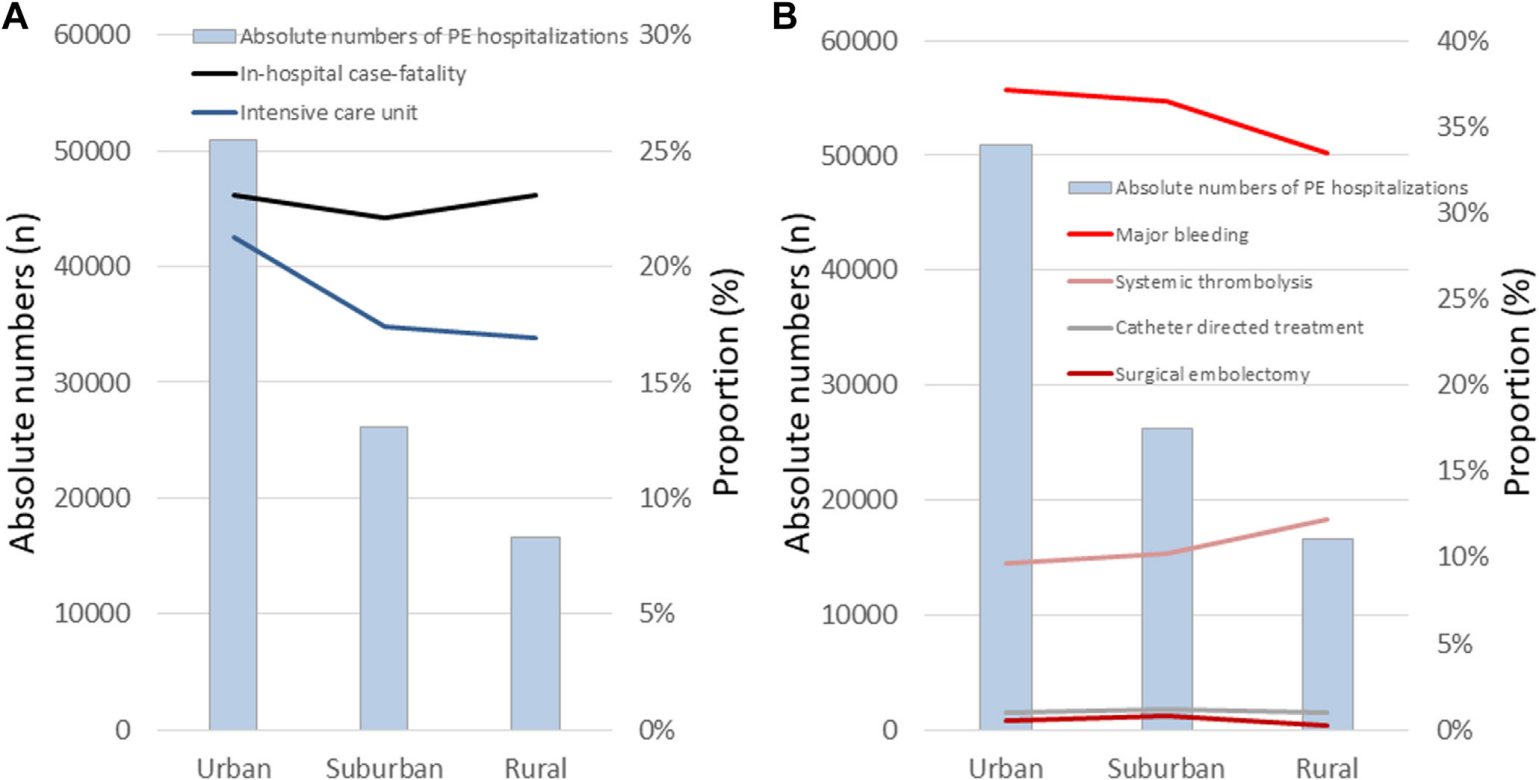
Regional trends regarding intensive care unit admission rates in pulmonary embolism (PE) patients as well as rates of case fatality, major bleeding, and use of reperfusion strategies of PE patients admitted to an intensive care unit.

In addition, we investigated regional differences regarding ICU admission, case fatality, major bleeding, and reperfusion strategies. While the majority of PE patients were treated in urban hospitals, the highest ICU admission rate was also observed for urban hospitals (Figure 4). While in-hospital case fatality rate was similar for patients treated in ICUs of urban, suburban, and rural hospitals, rate of major bleeding was lowest in rural hospitals, most likely through an early transfer of some of the hemodynamically compromised patients from rural to larger hospitals in urban areas such as university hospitals. Regarding regional trends of reperfusion treatment, systemic thrombolysis was slightly more often used in PE patients admitted to an ICU of rural hospitals (Figure 4).

## 4 Discussion

PE is a potentially life-threatening condition exhibiting the third most common cardiovascular cause of death [2,4,17]. The central pathomechanism leading to death in patients with acute PE is acute RV failure resulting in low systemic output with hemodynamic compromise/instability (intermediate high-risk and high-risk PE). In those patients with hemodynamic compromise, immediate reperfusion is recommended by the European and American guidelines [10,18]. In this context, the primary aim in the treatment of acute PE is to restore blood flow to the affected areas of the lungs by resolving/removing embolus mass and additionally preventing further clot formation and embolization [2,10,18]. Besides these aggressive reperfusion treatments, ICU admission is another important component regarding the adequate management of these hemodynamically compromised PE patients.

PE patients frequently require ICU treatment, most often driven by hemodynamic instability and/or severe hypoxemia [19,20]. The rate of PE patients admitted to ICU was 19.0% in Germany, higher than in Japan at 15.4% [21], but lower than reported data in the United States at 28.0% [22]. In our study, 51% of the patients admitted to a German ICU had a PE with imminent or present decompensation, and 24.3% had a hemodynamic instability, which is lower than the rates of 50.2% and 57.5% hemodynamic instability of PE patients admitted to an ICU in Japan [21] and Tunisia [23], respectively. These signs of hemodynamic compromise are strong and independent predictors for PE patients to be admitted to a German ICU. In addition, it is well known that patients who underwent reperfusion treatments are normally monitored in ICU [3,11,[24], [25], [26]].

While the median age of PE patients treated in German ICUs was 69 years, the median age of PE patients admitted to ICUs in Australia and New Zealand was 66.5 years [27] and those of ICU patients with PE in Saudi Arabia as well as Tunisia were substantially lower at 40.6 years [20] and 54.9 years [23], respectively. This finding is noteworthy in light of aging societies and an increasing incidence of PE with age [2,28]. These findings of higher median age in combination with a lower proportion of hemodynamic unstable PE patients admitted to German ICUs might reflect the higher number of ICU capacities in Germany in comparison with other countries [29]. In Germany, the sex distribution of ICU patients with PE was widely balanced (female sex 48.1%), and male sex prevailed in other countries [20,23].

Since PE patients in German ICUs were older, these patients showed an aggravated comorbidity profile compared with the younger patients in ICUs in other countries [20,23,27]. Especially, the rate of cancer was especially higher in German ICUs [27], which also supports the hypothesis that the ICU capacities are larger in Germany than in other countries with more frequent necessity of triage [29]. Our results demonstrated that the in-hospital case fatality rate was more than doubled when PE patients had to be admitted to an ICU, and this 2.5-fold risk was independent of age, sex, and comorbidities. The calculated case fatality rate of 22.7% was lower than in Tunisia (52.9%) [23] but higher than in Australia and New Zealand (7.7% at 30 days) [27] and Saudi Arabia (14%) [20], whereby the older age in German patients has to be taken into account when interpreting these results.

As expected, the length of in-hospital stay was more than doubled when ICU treatment was needed, and ICU admission was independently associated with a 3.6-fold risk of an in-hospital stay >10 days.

The key objective of our study was to identify independent risk factors other than hemodynamic compromise for ICU admission in acute PE. In this context, cardiovascular risk factors; the classical VTE risk factors such as surgery, stroke, and pregnancy, and cardiopulmonary; and renal comorbidities were independently associated with ICU admission. It is well known that COVID-19 is associated with PE development but also with aggravated outcomes in PE [29,30,31]. Taking together, our data demonstrated that an aggravated individual comorbidity profile is an important trigger for ICU admission, mirrored by the association between Charlson Comorbidity Index class and increased risk for ICU admission. This result of our study is in line with published literature indicating an important influence of comorbidity burden on the outcome of PE [[32], [33], [34], [35], [36], [37], [38]]. Although our study helps to identify PE patients with a more complicated course during the initial phase of PE [[39], [40], [41], [42], [43], [44], [45]], other associations regarding bleeding complications but also survival are of greater concern: it is of outstanding interest that PE patients admitted to an ICU had a more than 5-fold risk of developing major bleeding during hospitalization. In this context, all reperfusion strategies were afflicted with an elevated bleeding risk when administered in those PE patients admitted to an ICU, whereas the risk for major bleeding was markedly increased, especially related to surgical embolectomy. These findings are in line with previously published results revealing that usage of systemic thrombolysis was accompanied by increased occurrence of major bleeding [[46], [47], [48]], but CDT was also associated with increased risk of major bleeding [[49], [50], [51], [52]] compared with anticoagulant treatment [53]. The bleeding risk in CDT is substantially influenced by patient selection, treating more often PE patients who are at elevated risk for major bleeding to avoid the use of systemic thrombolysis and minimize bleeding events, which were expected to occur more commonly related to systemic thrombolysis rather than CDT. Since systemic thrombolysis and surgical embolectomy are established treatment options for acute PE with hemodynamic deterioration [18,54] and CDT—as an emerging treatment option for PE—is recommended for selected PE patients with decompensation [18,52,54], these treatments are beneficial for selected PE patients with existing or impending decompensation (high-risk or intermediate high-risk PE patients), but not for all PE patients and especially not for PE patients without decompensation (low-risk PE patients) [2,18,54]. These recommendations were in part supported by our study results. While systemic thrombolysis and surgical embolectomy were not independently associated with reduced case fatality in PE patients admitted to ICU regardless of PE severity, CDT was related to decreased case fatality in PE patients admitted to ICU with 0.8-fold risk to die independently of age, sex, and comorbidities. These data regarding effects of reperfusion treatments in PE patients treated in ICU support previously published study results by our research group for all PE patients and patients with severe PE in Germany, focusing on cost drivers in acute PE [55]. Our data underline once again the outstanding importance of optimal patient selection for the different reperfusion treatments. ICU admission without strong risk stratification regarding hemodynamic compromise in PE is not an adequate criterion for selection of patients who should be treated with reperfusion strategies. In addition, due to the fear of major bleeding, the established treatment strategy for decompensated PE patients of systemic thrombolysis was underused. Although approximately a quarter of the PE patients treated in ICU were hemodynamically unstable, systemic thrombolysis was used in only 10.3% and CDT in 1.1% of the patients. The use of these beneficial, life-saving, and recommended reperfusion treatments in decompensated PE patients is still unacceptably low in this real-world data of the German nationwide inpatient sample. Thus, the data of our present study, which are in accordance with previously published studies [2,8,47,56], indicate an underuse of these reperfusion treatments in decompensated PE and raise the claim to improve and optimize the management of patients with decompensated PE.

Summarizing these results, ICU treatment is an important element in stabilizing and monitoring PE patients as well as for advanced therapies with aggressive treatment strategies including reperfusion approaches. In addition, ICU treatments and ICU physicians play an important role in decision-making and guidance regarding adequate PE management. In most hospitals, ICU physicians are commonly involved in PE response team (PERT), which are increasingly implemented in hospitals to optimize the treatment of patients with acute PE and overcome the reservations and the therefrom resulting underuse of reperfusion treatments in patients with decompensated PE [2,3,24,57,58]. PERT brings together a team of specialists from different disciplines, comprising specialists in cardiology, pulmonology, hematology, vascular medicine, intensive care, cardiothoracic surgery, and (interventional) radiology [3,24]. The exact composition and operating mode of a PERT are not fixed and depend on the resources and also on the expertise available in each hospital for the optimization regarding the management of acute PE, but intensive care specialists are in the majority of cases included in the PERT [3,24].

### Limitations

The present study has some limitations. Due to the nature of ICD and OPS code–based study analysis of hospitalized patients, underreporting and undercoding are possible, and data on concomitant medication or laboratory markers are unavailable. Also, no follow-up evaluation is available since data are limited to the time frame of the in-hospital course. The data included in the German nationwide inpatient sample represent all population parts of Germany. However, information on the sociocultural determinants of health and race/ethnicity of the study population is unavailable in the data set provided by the RDC. Thus, the transferability of the study results to other populations might not be unaffectedly possible with certainty.

## 5 Conclusion

ICU treatment is an important element in the treatment of PE patients. Besides hemodynamic compromise, cardiovascular risk factors; the classical VTE risk factors such as surgery, stroke, and pregnancy; and cardiopulmonary as well as renal comorbidities were independent predictors of ICU admission. The necessity of ICU admission was afflicted by increased case fatality in acute PE.
